# What makes people decide who to turn to when faced with a mental health problem? Results from a French survey

**DOI:** 10.1186/1471-2458-7-188

**Published:** 2007-07-31

**Authors:** Viviane Kovess-Masféty, Delphine Saragoussi, Christine Sevilla-Dedieu, Fabien Gilbert, Agnieszka Suchocka, Nathalie Arveiller, Isabelle Gasquet, Nadia Younes, Marie-Christine Hardy-Bayle

**Affiliations:** 1 MGEN Foundation for Public Health; EA 4069 University of Paris 5, 3 square Max Hymans, 75748 Paris Cedex 15, France; 2 Versailles Hospital, 177 rue de Versailles, 78157 Le Chesnay Cedex, France; 3 Medical Policy Division (AP-HP), 3 avenue Victoria, 75184 Paris Cedex 04, France

## Abstract

**Background:**

The unequal use of mental health care is a great issue, even in countries with universal health coverage. Better knowledge of the factors that have an impact on the pathway to mental health care may be a great help for designing education campaigns and for best organizing health care delivery. The objective of this study is to explore the determinants of help-seeking intentions for mental health problems and which factors influence treatment opinions and the reliance on and compliance with health professionals' advice.

**Methods:**

441 adults aged 18 to 70 were randomly selected from the general population of two suburban districts near Paris and agreed to participate in the study (response rate = 60.4%). The 412 respondents with no mental health problems based on the CIDI-SF and the CAGE, who had not consulted for a mental health problem in the previous year, were asked in detail about their intentions to seek help in case of a psychological disorder and about their opinion of mental health treatments. The links between the respondents' characteristics and intentions and opinions were explored.

**Results:**

More than half of the sample (57.8%) would see their general practitioner (GP) first and 46.6% would continue with their GP for follow-up. Mental health professionals were mentioned far less than GPs. People who would choose their GP first were older and less educated, whereas those who would favor mental health specialists had lower social support. For psychotherapy, respondents were split equally between seeing a GP, a psychiatrist or a psychologist. People were reluctant to take psychotropic drugs, but looked favorably on psychotherapy.

**Conclusion:**

GPs are often the point of entry into the mental health care system and need to be supported. Public information campaigns about mental health care options and treatments are needed to educate the public, eliminate the stigma of mental illness and eliminate prejudices.

## Background

There has been major concern about the unequal use of mental health care, even in countries with universal coverage such as Canada [1] where, despite such coverage, people from higher socioeconomic backgrounds receive more psychiatric care than those from lower socioeconomic backgrounds for equivalent mental health problems.

In addition to universal coverage, mental health literacy has been established as an important factor in the provision of adequate care, not only for people to be able to recognize specific disorders, but also for them to believe help is possible and available. People need to have the right information and knowledge about the professionals available, as well as about the different types of treatment [2]. In turn, their beliefs seem to be influenced by the improvements made to the mental health care system, such as an increase in psychotherapies available and out-patient unlabeled treatments [3]. On the other hand, a huge amount of information has been disseminated among the public from non-scientific sources, such as newspapers and television [2]. This has led a majority of people to prefer psychotherapy and to disregard psychotropic drugs, in contradiction to most of the literature on outcome [2]. This trend has been observed in many different countries [4].

In developed countries, the general practitioner (GP) is most frequently the first contact [5-9] and plays an important role as an entry point into the system, followed by potential care from mental health specialists [10]. This is true even if the extent of referral to a specialist may significantly differ among countries [11]. Indeed, people suffering from mental health problems may turn to various providers to look for help, including GPs, mental health specialists – subdivided between medical (psychiatrists) and non-medical practitioners (psychologists and various psychotherapists) – or even social services or what is labeled as "alternative" medicine [11]. Moreover, some people prefer to rely on their family and friends, even when suffering from severe disorders [2,3].

Although the use of mental health care was found in Europe to be only partially related to healthcare provision [11], some system-related factors may have an impact on the pathway to mental health care [12,13]. For instance, in some health care systems, access to mental health providers is conditional on a visit to a GP who acts as a gatekeeper. Other systems are less strict. On the other hand, financial aspects can also play a role. If free-of-charge or inexpensive care is available, this can lead people to turn to one resource rather than another [14].

Moreover, features specific to each patient, such as the level of social support, have been reported as playing an important role in the different pathways taken towards mental health care [15,16]. However, specific types of mental disorders (i.e., for example social phobia) may have a major impact on the level of social support as well. Being female, having a higher level of education, together with a high level of social support, seem to produce the best ingredients to receive treatment according to evidence-based literature [17].

Improved knowledge of these factors is of relevance to mental health care planners when designing education campaigns, as well as for delivering care [18]. This study covers the determinants of help-seeking intentions for mental health problems: selecting primary care versus specialized mental health or relying on one's own network. In addition, our study focuses on exactly what influences people's opinions on the different treatments available, and their reliance on and compliance with their GPs' advice since these professionals have become a major focus for implementing mental health awareness campaigns. This study, which is part of a European project [19,20], is based on a phone survey of a general randomized population in two relatively wealthy suburbs in the Paris area, in a country where the density of professionals is high, the majority of mental health and primary care resources are available free-of-charge and access to care is virtually unrestricted.

## Methods

### Sample

The sample was constituted through telephone numbers randomly taken from the phone directories in two counties in the Paris suburbs (Yvelines and Essonne). Respondents were recruited between September 2001 and January 2002. One interviewee per household was selected using the Kish method [21]. Subjects included were aged 18 to 70, had lived in the county for at least six months and were not institutionalized. Eligible individuals were asked to give their verbal consent to participate in a telephone interview. Written consent was requested only of those participating in the follow-up survey. Ethical approval was not required for this project.

The response rate was 60.4% and the number of respondents was 441 persons. Among them, 25 people (5.7%) were excluded as they were detected as having one of the measured psychiatric diagnoses or had reported at least one contact with a provider for any mental health problem in the twelve previous months. Of the remaining 416 people, 4 did not answer the demographic questions, leading to a final sample of 412.

### Instruments

The questionnaire was based on the CIDI-SF [22] to detect depression and generalized anxiety disorder and the CAGE [23] questionnaire to detect alcohol abuse. Subjects not diagnosed as having any disorder and who did not declare that they had consulted for a mental health problem during the last twelve months, were asked in detail about their intention to consult in the event they felt they were suffering from a mental health problem and their opinion about mental health care options available.

**Figure 1:**
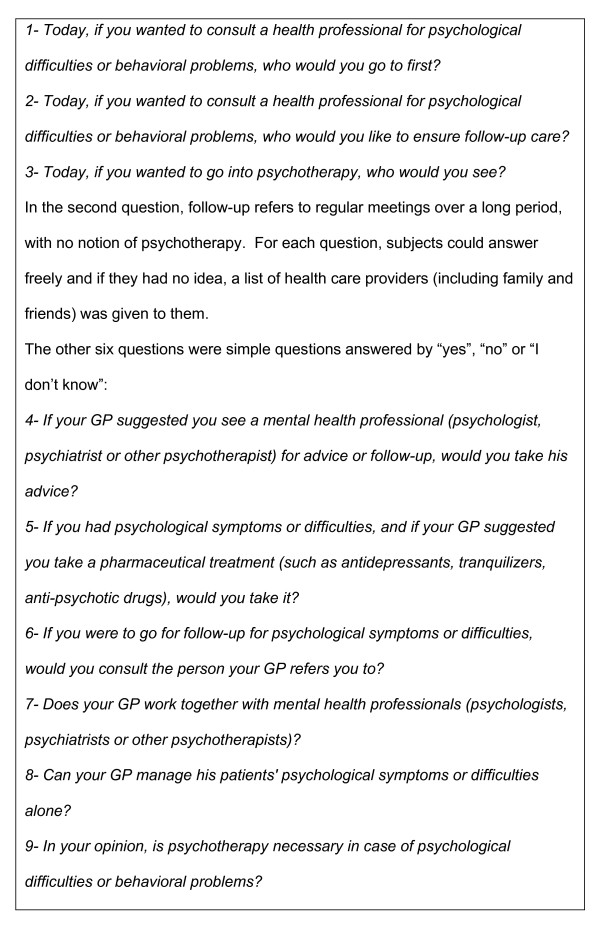
Questions concerning intentions and opinions toward help-seeking for psychological problems.

Nine questions concerning intentions and opinions towards help-seeking for psychological problems were especially designed for this study (Figure 1). The first three questions focused on the type of help people would seek in case of a mental health disorder. For each question, a list of health care providers was given to them. If necessary, the respondents were provided with definitions of mental health professionals (in order to avoid frequent confusion between psychiatrists who are medical doctors and psychologists who are not). The other questions were simple questions answered by "yes", "no" or "I don't know" concerning intentions and opinions towards GPs and psychotherapy.

The impact of social support on the subjects' intentions was evaluated using the Oslo 3-item social support scale [24], which has already proved to be feasible, reliable, and valid in other studies [25,26]. The questionnaire also covered basic demographic questions on gender, age, educational level, marital status and professional status.

### Statistical analysis

The differences between ratios were measured using Chi-square tests. The statistical analyses were performed using StataSE 9 software.

## Results

### Sample description

Table 1 shows the characteristics of the sample. The majority of the respondents were female (63.6%). The comparison of the characteristics between men and women revealed no significant difference on most of the variables, with the exception of professional status and social support. Women were less often employed and had a higher level of social support.

**Table 1:** Sample description

**Characteristics**	**Overall sample (N = 412)**		**Men (N = 150)**	**Women (N = 262)**	**p**
			
	**n**	**%**	**%**	**%**	
Gender					
Male	150	36.4			
Female	262	63.6			
Age					0.50
18–34	109	26.5	28.2	23.3	
35–49	142	34.5	34.4	34.7	
≥ 50	161	39.1	37.4	42.0	
Educational level					0.05
Primary, secondary* or vocational school	105	25.5	24.0	26.3	
Secondary school**	95	23.1	17.3	26.3	
University	212	51.5	58.7	47.3	
Marital status					0.06
Single	114	27.7	30.7	26.0	
Married	251	60.9	62.7	59.9	
Divorced or widowed	47	11.4	6.7	14.1	
Origin					0.37
French	366	88.8	90.7	87.8	
Foreign	46	11.2	9.3	12.2	
Professional status					< 0.01
Employed	265	64.3	70.0	61.1	
Retired	72	17.5	20.7	15.7	
Other	75	18.2	9.3	23.3	
Social support					0.02
Low	98	23.8	31.3	19.5	
Moderate	245	59.5	53.3	63.0	
High	69	16.8	15.3	17.6	

* = for students aged 11 to 14

** = for students aged 15 to 17

### Descriptive analyses

Descriptive analyses (Table 2) show that more than half of the sample (57.8%) would seek out a GP first in the event of psychological or behavioral difficulties, followed equally by a psychologist (15.1%) and a psychiatrist (14.6%). However, men and women differed on many aspects (p < 0.01). Women would turn more to GPs (60.7% vs. 52.7%) and psychologists (17.9% vs. 10.0%), whereas men would rather see a psychiatrist (18.0% vs. 12.6%) or their close friends and family (12.7% vs. 4.2%). When asked about long-term care, half of the sample would turn to a GP (46.6%) and in this case, no difference was identified between men and women (p = 0.15).

**Table 2:** Frequency of answers to the 9 questions about intentions and opinions toward help-seeking for psychological problems

	**Who would you go to first?**				**Who would you like to ensure follow-up care?**				**If you wanted to go into psychotherapy, who would you see?**			
	
			**p < 0.01**				**p = 0.15**				**p = 0.48**	
									
	**Overall Sample ** **(N = 412)**		**Men ** **(N = 150)**	**Women** ** (N = 262)**	**Overall Sample ** **(N = 412)**		**Men ** **(N = 150)**	**Women ** **(N = 262)**	**Overall Sample ** **(N = 412)**		**Men** ** (N = 150)**	**Women ** **(N = 262)**
	
	**n**	**%**	**%**	**%**	**n**	**%**	**%**	**%**	**n**	**%**	**%**	**%**
GP	238	57.8	52.7	60.7	192	46.6	44.7	47.7	92	22.3	22.0	22.5
Psychiatrist	60	14.6	18.0	12.6	69	16.8	19.3	15.3	92	22.3	22.0	22.5
Psychologist	62	15.1	10.0	17.9	81	19.7	14.7	22.5	96	23.3	21.3	24.4
Friends/Family	30	7.3	12.7	4.2	-	-	-	-	-	-	-	-
Other	15	3.6	4.7	3.1	43	10.4	13.3	8.8	70	17.0	21.3	14.5
Don't know	7	1.7	2.0	1.5	27	6.6	8.0	5.7	62	15.1	13.3	16.0

	**If your GP suggested you see a mental health professional, would you take his advice?**				**If your GP suggested you take a pharmaceutical treatment, would you take it?**				**Would you consult the person your GP refers you to?**			
	
			**p = 0.03**				**p = 0.57**				**p = 0.13**	
									
	**Overall Sample** ** (N = 412)**		**Men ** **(N = 150)**	**Women ** **(N = 262)**	**Overall Sample** ** (N = 412)**		**Men ** **(N = 150)**	**Women ** **(N = 262)**	**Overall Sample ** **(N = 412)**		**Men ** **(N = 150)**	**Women ** **(N = 262)**
	
	**n**	**%**	**%**	**%**	**n**	**%**	**%**	**%**	**n**	**%**	**%**	**%**

Yes	326	79.1	72.0	83.2	229	55.6	58.7	53.8	342	83.0	78.7	85.5
No	66	16.0	22.0	12.6	150	36.4	34.7	37.4	51	12.4	16.7	9.9
No opinion	20	4.9	6.0	4.2	33	8.0	6.7	8.8	19	4.6	4.7	4.6

	**Does your GP work together with mental health professionals?**				**Can your GP manage his patients' psychological symptoms or difficulties alone?**				**Is psychotherapy necessary in case of psychological difficulties or behavioral problems?**			
	
			**p = 0.71**				**p = 0.22**				**p = 0.12**	
									
	**Overall Sample ** **(N = 412)**		**Men ** **(N = 150)**	**Women ** **(N = 262)**	**Overall Sample ** **(N = 412)**		**Men ** **(N = 150)**	**Women ** **(N = 262)**	**Overall Sample ** **(N = 412)**		**Men ** **(N = 150)**	**Women ** **(N = 262)**
	
	**n**	**%**	**%**	**%**	**n**	**%**	**%**	**%**	**n**	**%**	**%**	**%**

Yes	82	19.9	22.0	18.7	114	27.7	24.0	29.8	334	81.1	77.3	83.2
No	80	19.4	19.3	19.5	208	50.5	50.0	50.8	41	10.0	14.0	7.6
No opinion	250	60.7	58.7	61.8	90	21.8	26.0	19.5	37	9.0	8.7	9.2

In response to the question, "Today, if you wanted to go into psychotherapy, who would you see?", the answers were equally divided among the different options. 15.1% did not know, 22.3% mentioned their GP (most likely with the idea that their GP would refer them to a therapist), followed by psychiatrists (22.3%) and psychologists (23.3%), who were mentioned approximately at the same rate. No difference between men and women was observed (p = 0.48).

The marked preference of the majority of our sample for GPs shows the high level of trust patients have in them. Indeed, 79.1% of the respondents were ready to see a mental health professional referred to them by their GP. However, we did find that women would actually take their GPs' advice more easily than men (83.2% vs. 72.0%; p = 0.03).

However, trusting a GP no longer seems to be the rule when it comes to psychotropic drug prescription: 36.4% of the sample stated they would not take a psychotropic drug prescribed by their GP and an additional 8% did not know what they would do. In the end, only 55.6% said "yes", they would take a psychotropic drug prescribed by their GP and once again, no difference was identified between men and women (p = 0.57). This distrust in pharmaceutical drugs goes hand-in-hand with a general approval of psychotherapy, since 81.1% agreed that psychotherapy was needed in the event of a psychological disorder, with again no difference between men and women as regards this opinion (p = 0.12).

Nearly thirty percent (27.7%) thought that GPs would be able to manage mental health disorders on their own. More than half (50.5%), however, thought their GP had to work in cooperation with other professionals, but most of the respondents were unaware if their GP worked together with mental health professionals (60.7%).

### Multiple regression analyses

In order to evaluate what motivated the respondents' choices, we conducted multiple logistic regression analyses, adjusted for gender, for each of the questions using demographic variables, together with social network intensity measures.

In response to the question, "Today, if you wanted to consult a health professional for psychological difficulties or behavioral problems, who would you go to first?", we looked initially at the probability of turning to a mental health provider versus a GP (Table 3). The results showed that older, as well as less educated people would turn more often to a GP, whereas those with a smaller social network favored mental health providers. When specific regressions were then performed for psychiatrists or psychologists versus a GP, respectively, age and social network were significant for psychologists, whereas educational level was significant for psychiatrists. When we subsequently looked at the probability of consulting a psychologist first versus a psychiatrist, our models showed that women would favor psychologists over psychiatrists (OR = 3.05; p = 0.01), together with people with a smaller social network (OR = 2.94; p = 0.02). Finally, when friends and family were compared to professional help, men clearly favored this option (OR = 3.60; p = 0.00) over professionals, compared to women.

**Table 3:** Factors influencing the likelihood of consulting first a psychiatrist or a psychologist versus a GP in case of psychological problems

	**Overall sample (N = 360)**			**Men (N = 121)**			**Women (N = 239)**		
	
	**β**	**OR**		**β**	**OR**		**β**	**OR**	
	
	**β SE**	**OR 95%CI**	**p**	**β SE**	**OR 95%CI**	**p**	**β SE**	**OR 95%CI**	**p**
Gender									
Male	0	1							
Female	-0.020 0.25	0.98 0.60–1.60	0.94						
Age			*0.01*			*0.48*			*0.03*
18–34	-0.004 0.30	1.00 0.55–1.80	0.99	0.126 0.57	1.13 0.37–3.44	0.82	-0.049 0.36	0.95 0.47–1.94	0.89
35–49	0	1		0	1		0	1	
≥ 50	-0.779 0.28	0.46 0.26–0.80	0.01	-0.499 0.47	0.61 0.24–1.52	0.29	-0.939 0.37	0.39 0.19–0.80	0.01
Educational level			*0.15*			*0.79*			*0.22*
Primary, secondary* or vocational school	-0.581 0.30	0.56 0.31–1.00	0.05	-0.339 0.49	0.71 0.27–1.87	0.49	-0.686 0.39	0.50 0.23–1.09	0.08
Secondary school**	-0.189 0.29	0.83 0.47–1.47	0.52	-0.149 0.58	0.86 0.28–2.69	0.80	-0.200 0.35	0.82 0.41–1.62	0.57
University	0	1		0	1		0	1	
Marital status			*0.93*			*0.98*			*0.80*
Single	0.105 0.29	1.11 0.63–1.96	0.72	-0.092 0.54	0.91 0.32–2.63	0.87	0.235 0.36	1.27 0.63–2.54	0.51
Married	0	1		0	1		0	1	
Divorced or widowed	-0.001 0.38	1.00 0.48–2.09	1.00	-0.064 0.76	0.94 0.21–4.15	0.93	0.064 0.44	1.07 0.45–2.54	0.88
Social support			*0.02*			*0.18*			*0.12*
Low	0.731 0.28	2.08 1.20–3.58	0.01	0.794 0.45	2.21 0.92–5.33	0.08	0.689 0.37	1.99 0.97–4.10	0.06
Moderate	0	1		0	1		0	1	
High	-0.066 0.32	0.94 0.50–1.77	0.84	-0.030 0.59	0.97 0.31–3.06	0.96	-0.125 0.40	0.88 0.41–1.92	0.75

* = for students aged 11 to 14

** = for students aged 15 to 17

None of these determinants seemed to influence the opinion regarding psychotherapy with a preferred provider (psychologist vs. psychiatrist), with the exception of younger respondents who preferred psychologists (OR = 2.31; p = 0.04), and the distrust of psychotropic medication. Men, however, would not follow their GP's advice as easily as women when it comes to consulting a mental health care provider, and people with a reduced social network would behave the same way (Table 4). Finally, women seemed to believe more than men in psychotherapy as being necessary in case of psychological problems (OR = 1.85; p > 0.05 but just at 0.08). Here the effect of social network is U-shaped: those in the middle were those who preferred to rely on psychotherapy the most.

**Table 4:** Factors influencing the likelihood of not taking the GP's advice to see a mental health care professional

	**Overall sample (N = 392)**			**Men (N = 141)**			**Women (N = 251)**		
	
	**β**	**OR**		**β**	**OR**		**β**	**OR**	
	
	**β SE**	**OR 95%CI**	**p**	**β SE**	**OR 95%CI**	**p**	**β SE**	**OR 95%CI**	**p**
Gender									
Male	0.649 0.29	1.91 1.09–3.37	0.03						
Female	0	1							
Age			*0.37*			*0.15*			*0.96*
18–34	0.522 0.40	1.69 0.78–3.66	0.19	1.134 0.60	3.11 0.95–10.11	0.06	-0.118 0.55	0.89 0.30–2.63	0.83
35–49	0	1		0	1		0	1	
≥ 50	0.369 0.35	1.45 0.73–2.86	0.29	0.679 0.54	1.97 0.69–5.65	0.21	0.034 0.48	1.03 0.41–2.63	0.94
Educational level			*0.19*			*0.49*			*0.22*
Primary, secondary* or vocational school	0.553 0.34	1.74 0.90–3.37	0.10	0.475 0.50	1.61 0.60–4.29	0.34	0.812 0.49	2.25 0.86–5.87	0.10
Secondary school**	0.496 0.35	1.64 0.82–3.29	0.16	0.542 0.54	1.72 0.59–4.98	0.32	0.629 0.49	1.88 0.71–4.93	0.20
University	0	1		0	1		0	1	
Marital status			*0.80*			*0.57*			*0.85*
Single	0.100 0.36	1.11 0.55–2.22	0.78	0.141 0.53	1.15 0.40–3.28	0.79	0.242 0.52	1.27 0.46–3.54	0.64
Married	0	1		0	1		0	1	
Divorced or widowed	-0.277 0.49	0.76 0.29–1.96	0.57	-1.079 1.11	0.34 0.04–2.97	0.33	-0.153 0.57	0.86 0.28–2.60	0.79
Social support			*0.03*			*0.79*			*< 0.01*
Low	0.733 0.31	2.08 1.14–3.81	0.02	0.303 0.46	1.35 0.55–3.35	0.51	1.258 0.43	3.52 1.51–8.20	< 0.01
Moderate	0	1		0	1		0	1	
High	-0.206 0.45	0.81 0.34–1.97	0.65	-0.016 0.66	0.98 0.27–3.56	0.98	-0.317 0.66	0.73 0.20–2.68	0.63

* = for students aged 11 to 14

** = for students aged 15 to 17

## Discussion

This study shows that the majority of a French sample from the Paris suburbs intends to consult a GP first for a mental health problem and would comply with their GP's advice if they suggest that a visit to a mental health care provider is necessary. Psychiatrists and psychologists are mentioned equally. Psychotherapies are considered as a necessity and almost half of the respondents distrust pharmaceutical drugs or are not sure if they will take them if prescribed. Age, gender, educational level and social network also have some influence on the intentions stated.

Concerning the preference of our sample for GPs, the French are identical to respondents in a number of developed countries. However, the percentage of people mentioning mental health care providers appeared to be higher than in the Michigan survey, where 13% mentioned a psychiatrist and 10% a psychologist [7] and in the German survey, where respectively 10.4% and 9.8% mentioned specialist care as their first choice in case of depression [27]. However, this may be related to a higher density of psychiatrists in France [28].

Our survey shows that people who report their intention to consult a GP initially, as well as for follow-up are older (at least 50 years old) and less educated. Psychiatrists were chosen more often by those with a higher level of education (university level versus secondary only). This trend has been described in previous studies on attitudes and intentions in the United States and the Netherlands [29,30], as well as in Germany [27]. Access to a psychiatrist may therefore depend on a certain level of education and age, since for older and less educated people, psychiatrists are linked more to mental illness and the stigma behind it.

Easy acceptance of a referral to a mental health care provider has not been found in previous literature, but could be compared to the high level of confidence reported in an Australian study [2]. However, this result is still surprising, since in France, GP referral to mental health care professionals was found to be the lowest in a study comparing six European countries [11]. This finding underlines that in France, the problem is due more to our GPs' attitudes and beliefs than their patients.

Being a man or a woman seems to have a high impact on whether or not a GP's advice to consult a mental health care provider is complied with. Women are two times more likely to follow this advice compared to men. In addition, women are more inclined to visit a psychologist and men more inclined to see a psychiatrist. This may be explained by a higher propensity of women to talk about their problems, whereas men may prefer what they see as a more medical approach, such as a visit to a psychiatrist. Based on this hypothesis, women are slightly more in favor of psychotherapy than men (p = 0.08). Compared to women, men more frequently mentioned their family and friends versus any provider and this may reflect reluctance to accept a mental health care approach.

Social support has been found to play a key role in the choice of a provider. Respondents with a low level of social support clearly favor psychologists versus GPs, but also psychologists versus psychiatrists. This could be interpreted as a desire to talk about psychological difficulties rather than taking a more medical approach. People who claim they have a poor social network are less likely to rely on their friends and family in case of problems which seems only logical and this applies especially to men, who generally prefer this type of help. Respondents who have a low level of social support stated they did not want to comply with a mental health referral from their GP, but this concerned women only and is consistent with the belief that psychotherapy is a better approach. However, this contradicts their preference to see a psychologist rather than a GP. This may be due to the fact that people with a limited social network clearly favor psychologists over psychiatrists and that respondents may have mixed up the two professionals when asked about a referral to a mental health care provider. Those who do not prefer psychotherapy to solve psychological problems benefit from strong social support compared to those who have a moderate level of social support. This may be interpreted as reliance on their own network to solve problems. However, it must be kept in mind that those who have the smallest social network are also those who have the highest number of symptoms [10].

Just as in other countries, French people would rather engage in psychotherapy than take psychotropic drugs [31,32]. People believe that psychiatric drugs are addictive, useless or solely useful for symptoms [33]. In France, this marked preference for non-pharmaceutical treatment over psychotropic drugs, however, is in contrast to the very high level of psychotropic drug consumption [34]. In France, 80% of psychotropic drug prescriptions and 70% of antidepressant prescriptions are drawn up by GPs and 20% of these prescriptions meet no real psychiatric diagnosis [35]. The pressure on GPs, who are poorly trained to perform psychiatric diagnoses and who are overwhelmed by the demand without enough time to respond, has been put forward as an explanation for this, along with a lack of relations with mental health care specialists, together with their tendency to prescribe drugs in a generous public health insurance system.

It is also noteworthy that in general, people do not favor psychiatrists (whose fees are paid for by public health insurance) over psychologists (whose fees are not reimbursed in the majority of cases). Barriers to seeking help from mental health care professionals seem to be more dependent on knowledge, beliefs and stigma than on financial resources, corroborating Bayer and Peay's work [36].

Admittedly, this study has several limitations. Our sample was rather small and skewed toward women, so it cannot be truly representative of the Paris suburban population, but we carefully performed analyses taking into account both genders separately. However, the men who agreed to participate in the survey may be different from those who refused and may be more prone to seeking psychiatric care. As respondents were selected from two Parisian suburbs, our sample could not be considered as representative of the French population either, although indeed this was not the aim. However, it may be that the high density of mental health care providers in the Parisian area had an influence on people's intentions. In addition, intentions may differ from what people will actually do in case of a problem. Certain studies, however, seem to establish that opinions on mental health care are structured based on availability and do contribute to what will happen [18,36].

## Conclusion

Considering the results of our study, efforts in mental health care planning should be aimed in two directions. The first would be to support GPs as the initial entry point into the mental health care system. This has many advantages. GPs are familiar with the behavior of their patients and people claim they would comply if their GP suggests they see a mental health professional, even though they would prefer to be cared for by their own GP. In this case, GPs could take care of minor psychological disorders and refer major problems to specialists. Thiswould also contribute to regulating the activity of our mental health care professionals. Thissolution goes back to the question of training GPs in psychiatry and that of the relationship between primary and secondary care systems, which could be enhanced through network approaches to make contacts easier and to reduce GPs' reluctance to call on mental health professionals [37]. The second would be to organize public information campaigns on mental health care and the difference between psychiatrists, psychologists and the various treatment options available in order to educate the public and reduce prejudices. Campaigns of this type have already been conducted in Great Britain and in Australia. In Great Britain, the Defeat Depression Campaign using the media as an information relay which took place between 1991 and 1996 led to a change of about 5–10% in terms of knowledge about mental health problems, antidepressants and intentions to consult health professionals [38,39]. These types of campaigns will be launched very soon in France.

## Abbreviations

CAGE Cut down, Annoyed, Guilty, Eye-opener

CIDI-SF Composite International Diagnostic Interview-Short Form

GP General Practitioner

## Competing interests

The author(s) declare that they have no competing interests.

## Authors' contributions

VKM participated in the conception and design of the study and in the acquisition and interpretation of data, and extensively revised the draft of the article. DS participated in the analysis and interpretation of data, and drafted the article. CSD participated in the extensive revision of the draft of the article. FG participated in the analysis and interpretation of data. AS made critical revisions to the manuscript. NA did the literature review and reviewed the manuscript. IG participated in the conception and design of the study and in the acquisition of data, and reviewed the manuscript. MCHB participated in the acquisition of data and reviewed the manuscript.

## Pre-publication history

The pre-publication history for this paper can be accessed here:


